# Simulation of Natural Convection with Sinusoidal Temperature Distribution of Heat Source at the Bottom of an Enclosed Square Cavity

**DOI:** 10.3390/e26040347

**Published:** 2024-04-19

**Authors:** Min Zeng, Zhiqiang Wang, Ying Xu, Qiang Ma

**Affiliations:** School of Mechanical and Vehicle Engineering, Nanchang Institute of Science and Technology, Nanchang 330108, China; cengmin@stu.ncpu.edu.cn

**Keywords:** natural convection, lattice Boltzmann method, sinusoidal temperature, machine learning

## Abstract

The lattice Boltzmann method is employed in the current study to simulate the heat transfer characteristics of sinusoidal-temperature-distributed heat sources at the bottom of a square cavity under various conditions, including different amplitudes, phase angles, initial positions, and angular velocities. Additionally, a machine learning-based model is developed to accurately predict the Nusselt number in such a sinusoidal temperature distribution of heat source at the bottom of a square cavity. The results indicate that (1) in the phase angle range from 0 to π, *Nu* basically shows a decreasing trend with an increase in phase angle. The decline in *Nu* at an accelerated rate is consistently observed when the phase angle reaches 4π/16. The corresponding *Nu* decreases as the amplitude increases at the same phase angle. (2) The initial position of the sinusoidal-temperature-distributed heat source *L_c_* significantly impacts the convective heat transfer in the cavity. Moreover, the decline in *Nu* was further exacerbated when *L_c_* reached 7/16. (3) The optimal overall heat transfer effect was achieved when the angular velocity of the non-uniform heat source reached π. As the angular velocity increases, the local *Nu* in the square cavity exhibits a gradual and oscillatory decline. Notably, it is observed that *Nu* at odd multiples of π surpasses that at even multiples of π. Furthermore, the current work integrates LBM with machine learning, enabling the development of a precise and efficient prediction model for simulating *Nu* under specific operational conditions. This research provides valuable insights into the application of machine learning in the field of heat transfer.

## 1. Introduction

Natural convection in a square cavity is widely applied in various engineering disciplines, such as solar thermal receivers, the cooling of electronic components, nuclear reactors, biomedical applications, battery thermal management, and other relevant engineering domains [1,2,3,4,5,6]. Therefore, numerous scholars have extensively investigated and scrutinized natural convection phenomena across diverse scenarios.

Several scholars have conducted investigations on natural convection phenomena with varying working mediums. Wei et al. [7] adopted the lattice Boltzmann method to investigate the natural convection heat transfer of air in the turbulent flow within a cavity. The analysis revealed an increase in *Ra* and a corresponding increase in the rate of natural convection, with sufficient turbulence being achieved when *Ra* > 10^9^. Javaherdeh et al. [8] investigated the impact of a magnetic field on the heat transfer characteristics of nanofluids flowing between cold and hot corrugated walls, revealing a local *Nu* drop near the hot wall and a subsequent reduction in heat transfer efficiency. Several scholars have conducted research on natural convection in cavities of varying geometries. Zhu et al. [9], Huelsz et al. [10] and Li et al. [11] conducted an analysis on the natural convection phenomenon in a square cavity at various inclination angles, and concluded that *Nu* in the square cavity exhibits a power law relationship with *Ra* under specific inclination angles. Tian et al. [12] conducted an analysis of the natural convection process in a cavity with a sinusoidal wall surface under various amplitudes and found that the maximum value of *Nu* and optimal heat transfer occurred when the amplitude was 3 mm and wave number was 2. Uddin et al. [13] investigated the impact of vertical wall waveform on heat transfer and flow in the cavity, concluding that surface undulations can augment vortex formation and enhance thermal exchange. Jain et al. [14] investigated the heat transfer and flow characteristics of power law nanofluids in vertically curved wavy walls. The results indicated that the average *Nu* increases with increasing values of *Pr*, *Ra*, and aspect ratio, while decreasing with the increasing power law index and surface rippling degree. Akbarzadeh et al. [15] investigated the phenomenon of natural convection in a trapezoidal cavity filled with nanofluids and demonstrated that enhancing the volume rate of nanoparticles and adjusting the inclination angle of the trapezoidal cavity wall can effectively enhance convective heat transfer characteristics within the cavity. Ren et al. [16] conducted a simulation study on natural convection heat transfer in a high closed square cavity, revealing the presence of a well-defined center symmetric structure in both the temperature and velocity fields. The augmentation of *Ra* led to a heightened natural convection, thereby resulting in an amplified *Nu*. Furthermore, several scholars have conducted investigations on the phenomenon of natural convection occurring on a heated wall surface subjected to various heating methods. He et al. [17] employed the lattice Boltzmann method to simulate the natural convection phenomenon occurring under the local uniform heating mode at the bottom of a porous square cavity. Their analysis revealed that both the location and size of the high-temperature heat source exert significant influence on convective heat transfer. Additionally, the partially heated turbulent natural convection in a cubic cavity containing nanoparticles was investigated by Lafdaili et al. [18]. The results demonstrated that the presence of nanoparticles significantly enhanced convective heat transfer, while the location of heating exerted a notable influence on natural convection heat transfer.

To summarize, natural convection is widely distributed and holds significant importance in various engineering applications. However, limited research has been conducted on the sinusoidal-temperature-distributed heat source along the heating wall in the natural convection phenomenon. Therefore, LBM is employed in the present paper to investigate the natural convection in a square cavity with non-uniform heating at its bottom surface. The temperature distribution of the heat source is characterized by sinusoidal variations with different amplitudes, phase angles, and angular velocities. The main research content of this work is organized into four sections: (I) the effect of the amplitude of the sinusoidal-temperature-distributed heat source on convective heat transfer characteristics; (II) the influence of the initial location of the sinusoidal-temperature-distributed heat source on convective heat transfer characteristics; (III) the influence of the angular velocity of the sinusoidal-temperature-distributed heat source at high temperatures on convective heat transfer characteristics; (IV) the application of machine learning to the natural convection of sinusoidal-temperature-distributed heat sources, which can be applied in the field of the thermal design of lithium battery pack heat dissipation.

## 2. Numerical Simulation Method

### 2.1. Physical Model

**Figure 1:**
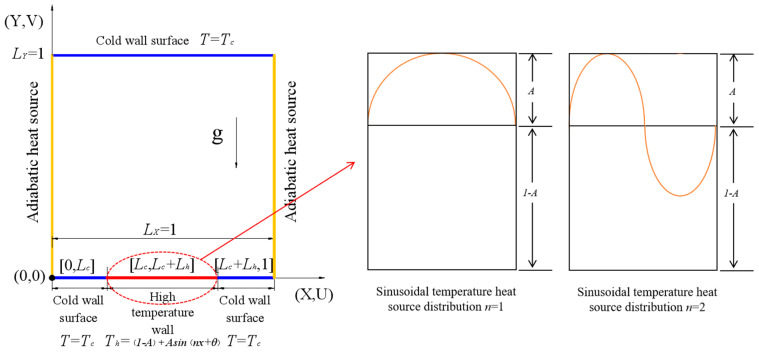
Convection heat transfer model of sinusoidal-temperature-distributed heat source.

Figure 1 shows the physical model of convection heat transfer with a non-uniform heat source. The geometric shape is a square cavity and the dimensionless length is *L_X_* = *L_Y_* = 1, where the left and right edges are adiabatic wall $\frac{\partial T}{\partial X}=\frac{\partial T}{\partial Y}=0$. The upper wall *T* = *T_C_* is low-temperature and constant-temperature wall *T*, corresponding to dimensionless temperature 0; the lower wall [*L_c_*, *L_c_* + *L_h_*] is a non-uniform heat source, and the dimensionless temperature distribution is *T* = (1 − *A*) + *Asin*(*nx* + *θ*). [0, *L_c_*] and [*L_c_* + *L_h_*, 1] are regarded as low temperature, and the wall temperature distribution is *T* = *T_C_*.

### 2.2. Governing Equation

In this paper, it is assumed that the fluid is incompressible and the thermal physical parameters are constant. The macroscopic governing equation is shown as follows:

(1) ∇·V=0

(2) $$\rho \frac{\partial V}{\partial t}+\rho \left(V\cdot \nabla \right)V=-\nabla p+\mu \nabla^{2}V+F$$

(3) $$\frac{\partial T}{\partial t}+V\cdot \nabla T=\nabla \left(\alpha \nabla T\right)$$

where (1)–(3) are the continuity equation, momentum equation and energy conservation equation, respectively. Where V, p, T are the macroscopic velocity, pressure and temperature of flow field, and $F=\left(0,\rho g\beta \left(T-T_{c}\right)\right)$. The Boussinesq hypothesis is adopted to deal with the density change, so as to study the natural convection phenomenon [19]. ρ, μ, α, g are the fluid density, hydrodynamic viscosity, thermal diffusion coefficient and acceleration of gravity, respectively.

The dimensionless expression of the macroscopic governing equation is as follows:

(4) $$\nabla \cdot V^{\prime }=0$$

(5) $$\frac{\partial V^{\prime }}{\partial t^{\prime }}+\left(V^{\prime }\cdot \nabla \right)V^{\prime }=-\nabla P^{\prime }+Pr\nabla^{2}V^{\prime }+RaPrT^{\prime }$$

(6) $$\frac{\partial T^{\prime }}{\partial t^{\prime }}+V^{\prime }\cdot \nabla T^{\prime }=\nabla^{2}T^{\prime }$$

where the characteristic scales of dimensionless length $\left(X,\;Y\right)$, velocity $V^{\prime }$, time $T^{\prime }$, pressure $P^{\prime }$, and temperature $T^{\prime }$ are L, α/L, $L^{2}/\alpha$, $\rho \alpha^{2}/L^{2}$, $\left(T_{h}-T_{c}\right)$, respectively. Additionally, the dimensionless parameters *Ra* and *Pr* are denoted as $Ra=\frac{g\beta \Delta TL^{3}}{\alpha \mu }$ and $Pr=\frac{\mu }{\rho \alpha }$, respectively.

### 2.3. Lattice Boltzmann Method Model

The lattice Boltzmann method (LBM) is different from the traditional Computational Fluid Dynamics method (CFD). LBM is a numerical method based on space, time and velocity discretization, and is a solution of the continuous Boltzmann equation, which has the advantages of the simple processing of boundary conditions, effective parallelism and easy programming [20].

The BGK-LBM model is employed in this paper due to its simplicity and ease, allowing for the linearization of the Boltzmann equation and thereby simplifying the solution process [21]. Therefore, the BGK-LBM model is adopted to simulate a natural convection process in this paper, wherein model D2Q9 is adopted for the flow field and temperature field [22]; the evolution process is as follows:

(7) $$f_{a}\left(x+\Delta x,t+\Delta t\right)=\left[1-w_{m}\right]f_{a}\left(x,t\right)+w_{m}feq_{a}\left(x,t\right)+F_{a}$$

(8) $$feq_{a}=\omega_{a}\rho \left(x,t\right)\left[1+\frac{c_{a}\cdot u}{cs^{2}}+\frac{{\left(c_{a}\cdot u\right)}^{2}}{2cs^{4}}-\frac{u^{2}}{2cs^{2}}\right]$$

(9) $$\rho =\sum f_{a}$$

(10) $$\rho u=\sum f_{a\;}c_{a}+\frac{\Delta t}{2}F_{a}$$

where $f_{a\;}$ and $f_{a}^{eq}$ are the velocity distribution function and equilibrium velocity distribution function, respectively. Additionally, $w_{m}$ is the collision frequency of the velocity field; wm=10.5+3μ. This paper analyzes the natural convection caused by density change, and the external force term $F_{a}=\frac{3\omega_{a}g\beta c_{a}\left(T-T_{c}\right)}{T_{h}-T_{c}}$.

(11) $$H_{a}\left(x+\Delta x,t+\Delta t\right)=\left[1-w_{\alpha }\right]H_{a}\left(x,t\right)+w_{\alpha }H_{a}^{eq}\left(x,t\right)$$

(12) $$H_{a}^{eq}\left(x,t\right)=\omega_{a}T\left(x,t\right)\left[1+\frac{c_{a}\cdot u}{cs^{2}}\right]$$

(13) $$T=\sum H_{a}$$

where $H_{a}$ and $H_{a}^{eq}$ are the temperature distribution function and equilibrium temperature distribution function, respectively. Moreover, $w_{a}$ is the collision frequency of temperature; wa=10.5+3α.

The weight coefficient is shown as follows:

(14) $$\omega_{a}=\left\{\begin{array}{c} 4/9\;\;\;\;a=0\\ 1/9\;\;\;\;\;\;\;\;\;a=1\sim 4\\ \;1/36\;\;\;\;\;\;a=5\sim 8 \end{array}\right.$$

The lattice velocity distribution in the D2Q9 model is as follows [22]:

(15) $$c_{a}=\left\{\begin{array}{c} \left(0,\;0\right)\;\;\;\;\;\;\;\;\;\;\;\;a=0\\ c\left(\cos\left[\left(a-1\right)\frac{\pi }{2}\right],\cos\left[\left(a-1\right)\frac{\pi }{2}\right]\right)\;\;\;\;\;\;\;\;\;\;\;\;a=1\sim 4\\ \sqrt{2}c\left(\cos\left[\left(2a-1\right)\frac{\pi }{4}\right],\sin\left[\left(2a-1\right)\frac{\pi }{4}\right]\right)\;\;\;a=5\sim 8 \end{array}\right.$$

## 3. Results and Discussion

### 3.1. Numerical Model Verification

**Figure 2:**
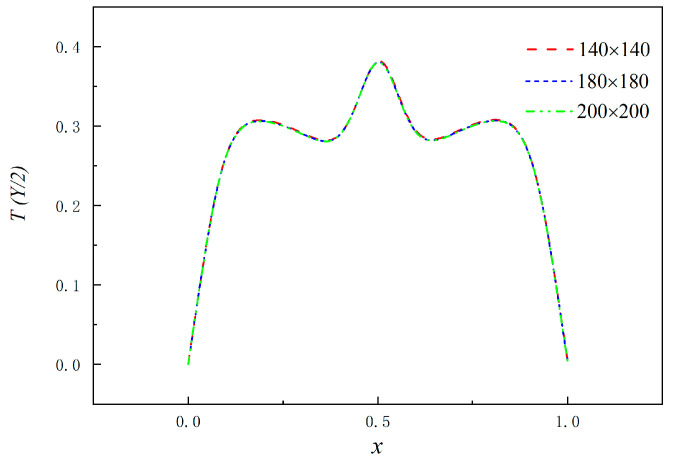
Grid independence verification of numerical models.

The current study validates the precision of grid distribution and confirms the accurate performance of the selected model. Firstly, based on Corvaro’s experimental conditions (*Pr* = 0.71, *Ra* = 2.02 × 10^5^) and boundary conditions, the temperature distributions at the midline position and the *Nu* of the heat source were compared under different grid distributions (140 × 140, 180 × 180, 200 × 200). The left and right temperature boundary is the low-temperature boundary, and the upper boundary is the adiabatic boundary. The lower boundary consists of a constant temperature heat source for the heat source part, while the remaining portion is characterized by an adiabatic boundary. By comparing the temperature distribution at the position of the horizontal midline, as depicted in Figure 2, it becomes challenging to discern intuitively any noticeable differences among the temperature distributions under the three grid distributions. However, the Nu of the heat source in the three cases is compared with Corvaro’s formula $Nu=0.5478Ra^{0.1998}$ [23]. It is observed that for a grid distribution of 140 × 140, there is a relatively larger relative error of 6.22%, while when the grid distribution is 180 × 180, the relative error decreases to 3.88%, which differs by less than 3.07% from the relative error obtained with a grid distribution of 200 × 200. The results are basically consistent, so as to ensure the simulation accuracy and save computing resources. Therefore, this paper selects 180 × 180 mesh precision for numerical simulation (Table 1).

**Figure 3:**
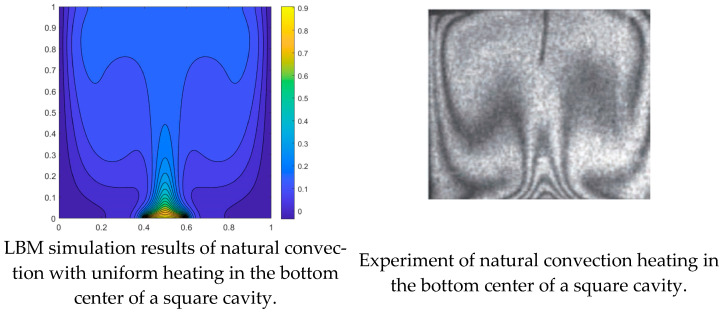
Comparison of numerical simulation results (**left**) and experimental temperature distribution [21] (**right**).

In order to ensure the accuracy of the simulation results, the BGK-LBM model was used to simulate and compare the natural convection results in a square chamber with Corvaro’s experiment with the same parameters (*Pr* = 0.71, *Ra* = 2.02 × 10^5^), as shown in Figure 3. The isotherm distribution of the numerical simulation and experimental results was in good agreement, thus verifying the accuracy of the model established in this paper.

### 3.2. Influence of Amplitude A of Sinusoidal-Temperature-Distributed Heat Source at High Temperature on Heat Transfer Characteristics of Natural Convection in Cavity

The characteristics of natural convective heat transfer in a square cavity can be ascertained by the Nusselt number at the boundary of the heat source [24], $Nu=-\frac{1}{L_{h}}\int_{L_{c}}^{L_{c}+L_{h}}\frac{\partial T}{\partial Y}{|}_{Y=0}dx$.

The influence of the amplitude *A* of heat source on convective heat transfer is investigated in this section. When *Pr* = 0.71 and *Ra* = 10^5^, the temperature distribution for a non-uniform heat source can be described by the function *T* = (1 − *A*) + *Asin*(*nx* + *θ*). The boundary length of heat source with a high-temperature non-uniform distribution is *L_h_* = 1/2, and the initial position is *L_c_* = 1/4. The natural convection process in a square cavity with *n* = π, *A* = 0.1, 0.2, 0.3, and 0.4 is analyzed in this section. The temperature distribution slope along the heat source increases with a larger magnitude of amplitude *A*, while it decreases with a smaller magnitude of amplitude *A*, as the temperature distribution of the heat source part is influenced by *A*.

**Figure 4:**
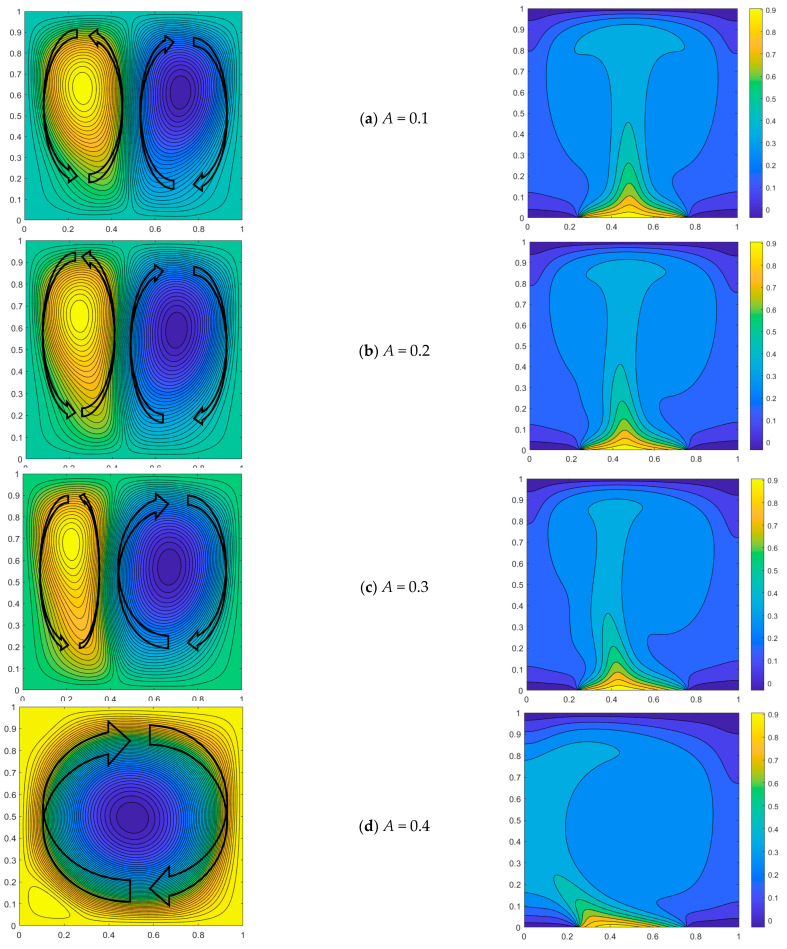
Streamline distribution (**left**) and temperature field distribution (**right**) for different A (*Ra* = 10^5^, *n* = π, *θ* = 2π/16).

When *Ra* = 10^5^ and *n* = π, increasing the amplitude results in a higher slope of the temperature sine distribution on the heat source, thereby amplifying the influence of heat source asymmetry and favoring the formation of the primary vortex within the cavity. For instance, at a phase angle of 2π/16, the heat source exhibits an uneven temperature distribution with a slightly higher left side and lower right side. When the amplitude *A* = 0.1, the non-uniform temperature difference of the heat source is 0.076, and the size of the two vortices formed in the flow field is approximately equal, with a slightly larger vortex observed on the right side but not significantly prominent. When the amplitude *A* reaches 0.2, the non-uniform temperature difference of the heat source attains a value of 0.153, thereby augmenting the impact of temperature difference on density within the flow field and leading to pronounced compression exerted by the right vortex onto the left vortex. When *A* increases to 0.4, the non-uniform temperature difference of the heat source reaches 0.306, and its non-uniformity further intensifies. The lift on the left side significantly surpasses that on the right side, resulting in an increased density difference. Consequently, the right vortex exerts additional pressure on the left vortex, leading to the emergence of a dominant main vortex (Figure 4) that occupies nearly the entire flow field.

**Figure 5:**
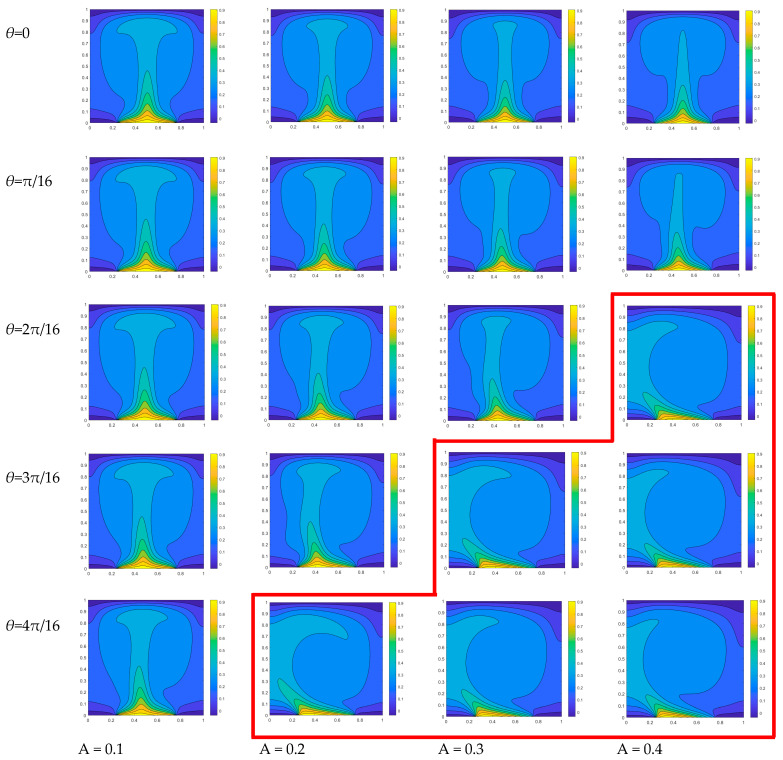
Temperature distribution diagram of high-temperature non-uniform heat source distributed at different phase angles *θ*.

When *A* = 0.1 and phase angles *θ* = 0, the isothermal temperature distribution in the square cavity exhibits symmetry, resulting in the generation of two vortices of equal size within the flow-field distribution diagram. When the phase angles increase to 4π/16, the maximum temperature in the boundary of the sinusoidal-temperature-distributed heat source shifts towards the left, resulting in a larger temperature difference between the left and right parts of the square cavity. The isotherm with a higher temperature exhibits a negligible deviation towards the left, while the vortex in the flow field on the right expands towards the left and marginally compresses the vortex on the left, as illustrated in Figure 5.

The relatively symmetrical temperature field is primarily attributed to the small amplitude *A*, which results in an inconspicuous uneven distribution of temperature in the heat source region and a negligible temperature difference. Additionally, there is no distinct density variation between the left and right parts.

When the amplitude *A* = 0.2, there is an observed increase in the slope of the temperature distribution within the heat source region. When the phase angle is equal to zero, the isotherm distribution of the temperature field remains symmetrical, resulting in a relatively high temperature at the center. Consequently, two symmetrical primary vortices will form within the flow field. As the phase angles increase, a gradual temperature asymmetry emerges with the left side of the heat source exhibiting higher temperatures compared to the right side at the central symmetric position. Consequently, an increasingly pronounced temperature difference arises and leads to a progressive compression of the vortex on the left by its counterpart on the right. When *θ* = 4π/16, the right vortex in the cavity fully occupies the space. The enhancement of temperature heterogeneity in the heat source is further pronounced when amplitude *A* increases to 0.3. At the same phase angle, the non-uniformity of the temperature field becomes more pronounced, with a noticeable inclination in the high-temperature isotherm towards the right side. Consequently, this leads to a reduced density of fluid on the right compared to that on the left within the flow field, resulting in a more prominent squeezing effect exerted by the right vortex onto the left vortex. When *θ* = 3π/16, the flow field retains only a single dominant vortex. When *A* reaches 0.4, the phase angle of the flow field forming the primary vortex decreases solely at *θ* = 2π/16. As depicted by the red step line in Figure 5, a single dominant vortex is observed within each square cavity below the red line, wherein the phase angle associated with this primary vortex decreases as the amplitude value increases.

**Figure 6:**
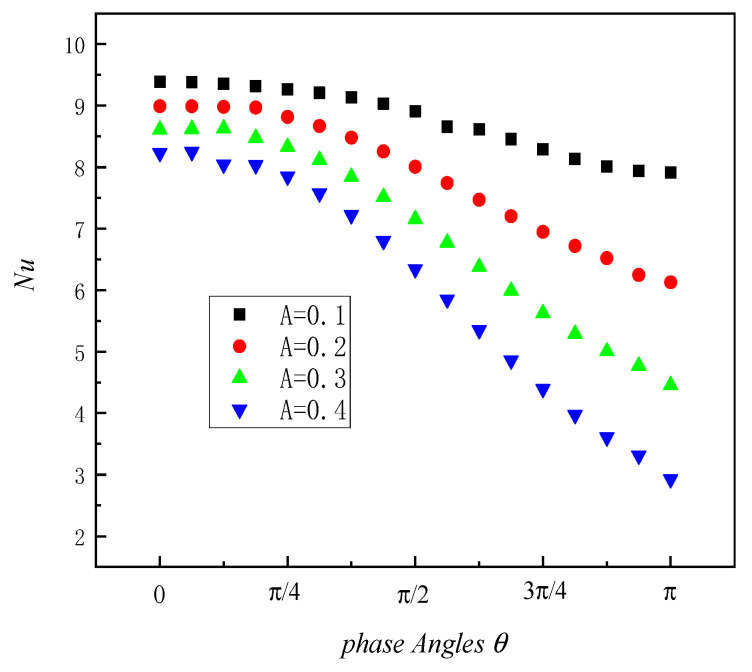
Local Nu varies with phase angle under different amplitudes *A*.

The variation in the local *Nu* of the heat source with the phase angle under different amplitudes is analyzed, which serves as a crucial parameter for evaluating the convective heat transfer effect, as depicted in Figure 6. Regarding the phase angle range from 0 to π, *Nu* basically shows a decreasing trend with an increase in the phase angle. The decline in *Nu* at an accelerated rate is consistently observed when the phase angle reaches 4π/16. The corresponding *Nu* decreases as amplitude *A* increases at the same phase angle. The *Nu* of the large amplitude value *A* exhibits a more pronounced decrease with an increase in the phase angle.

**Figure 7:**
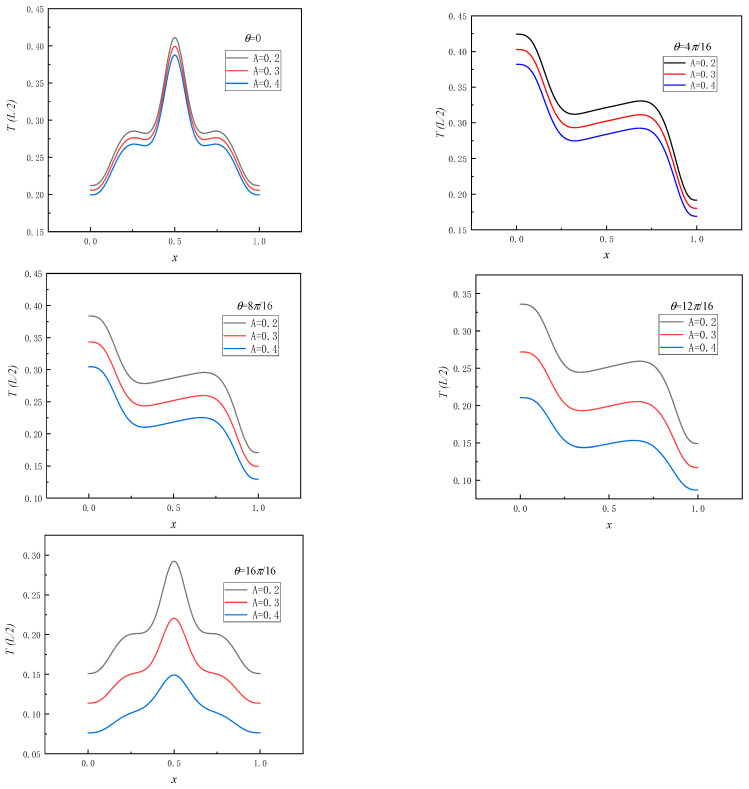
Temperature distribution curve of center line position in square cavity at different phase angles *θ*.

To investigate the convective heat transfer characteristics in a square cavity, focusing on temperature distribution, this section presents the variation in temperature along the central line for different phase angles, as illustrated in Figure 7. It can be seen from the figure that as the phase angle increases, the temperature distribution at the center line position in the square cavity gradually transitions from left–right symmetry to asymmetry and then reverts back to symmetry. The temperature at the same location decreases as amplitude *A* increases. Simultaneously, the phase angle significantly influences the maximum temperature position of the center line of the square cavity. The maximum temperature gradually decreases as the phase angle increases, while the temperature difference between different amplitude values is observed to increase.

### 3.3. Influence of Initial Position Variation in Sinusoidal-Temperature-Distributed Heat Source on Convective Heat Transfer Characteristics

In this section, the convective heat transfer conditions are investigated to examine the influence of the initial position of a sinusoidal-temperature-distributed heat source on its effect, considering a heat source length of 1/2 and temperature distribution *T* = (1 − *A*) + *Asin*(*nx* + *θ*). The analysis focused on different initial positions, namely, 1/4, 5/16, 3/8, 7/16, 1/2, respectively.

**Figure 8:**
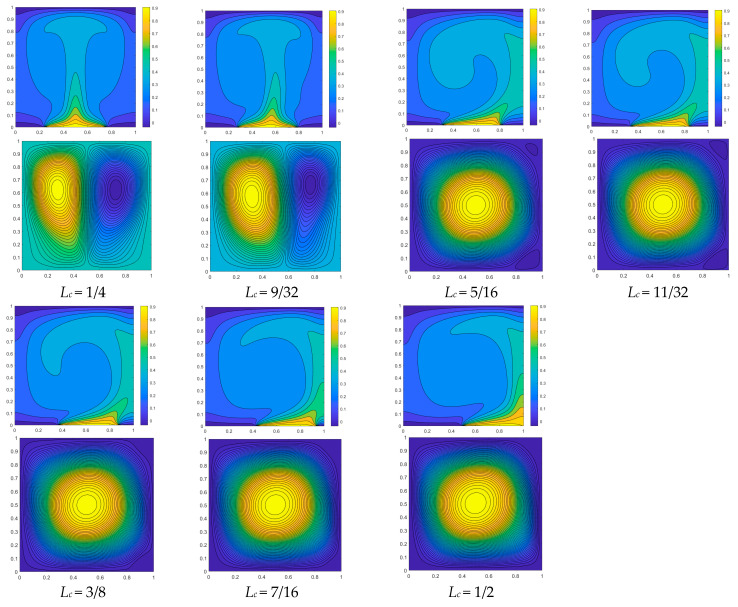
Comparison of the isotherm diagram (**up**) and flow field (**bottom**) of convective heat transfer with an initial position variation in heat source with *Ra* = 10^5^, *A* = 0.1, and *n* = π.

The flow field and isotherm diagram of convective heat transfer with *Ra* = 10^5^, *A* = 0.1, and *n* = π are depicted in Figure 8, illustrating the impact of the varying initial positions of the high-temperature heat source. The temperature field distribution exhibits symmetry when the initial position of the heat source *L_c_* = 1/4 is considered, as depicted in Figure 8. Additionally, two symmetrical vortices are observed within the flow field. When the heat source is initially positioned at *L_c_* = 9/32, a higher temperature gradient is observed on the left side of the heat source compared to the right side. As a result, the right side exhibits lower density compared to the left side, leading to the suppression of the vortex on the right by the dominant vortex on the left. When the heat source is initially positioned at *L_c_* = 5/16, a higher temperature gradient is observed on the left side of the heat source compared to the right side, leading to flow-field instability. The right vortex ascends due to buoyant lift and is compressed by the left vortex, resulting in the left vortex becoming the dominant one, as depicted in Figure 8. The temperature gradient on the right side of the heat source continues to decrease as the initial position of the hot source *L_c_* moves to 7/16, albeit inconspicuously. Therefore, the heat transfer effect is marginally diminished. When the initial position shifts to 1/2, a significant decrease in the temperature gradient of the heat source is observed, leading to a continuous decline in heat transfer capacity. This phenomenon can be attributed to the direct contact between the right end of the heat source and the adiabatic wall, as well as the elimination of heat dissipation through the cold wall. The flow field still exhibits a dominant left vortex, while the right vortex remains confined to the upper right corner with negligible alterations, as shown in Figure 8.

**Figure 9:**
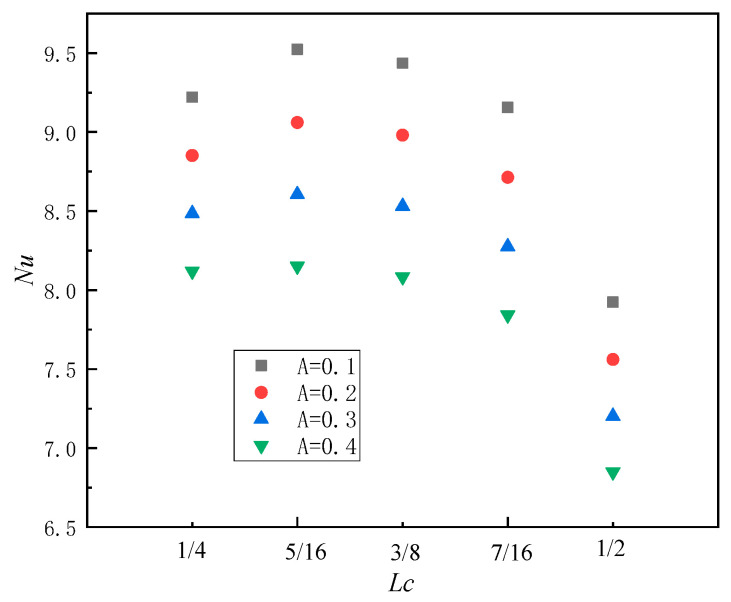
Local *Nu* in the square cavity varies with the initial position of sinusoidal-temperature-distributed heat source (*Ra* = 10^5^, *n* = π).

In order to comprehensively investigate the variation law of a sinusoidal-temperature-distributed heat source in a square cavity with respect to its initial position, the current work also examines the characteristics of local *Nu* in the square cavity (*Ra* = 10^5^, *n* = π) under different amplitude changes associated with the initial position of the sinusoidal-temperature-distributed heat source. When the amplitude *A* = 0.1, the local *Nu* initially increases to a maximum value of 9.52 as the heat source moves towards the right around *L_c_* = 5/16, and subsequently exhibits a continuous decrease, as illustrated in Figure 9, which corresponds to the conclusion in the previous section. Simultaneously, within the range of initial heat source positions from 1/4 to 7/16, there is negligible variation in the *Nu* value; however, when *A* = 0.1, a marginal change of 3.85% in the *Nu* number is observed. Subsequently, it slightly decreases with increasing amplitude and reaches a value of 3.79% at *A* = 0.4. When *L_c_* reaches 1/2, *Nu* exhibits a discernible decrease by approximately 13%, primarily attributed to a significant reduction in the temperature gradient near the right section of the heat source. This finding aligns with the conclusion presented in Figure 8.

**Figure 10:**
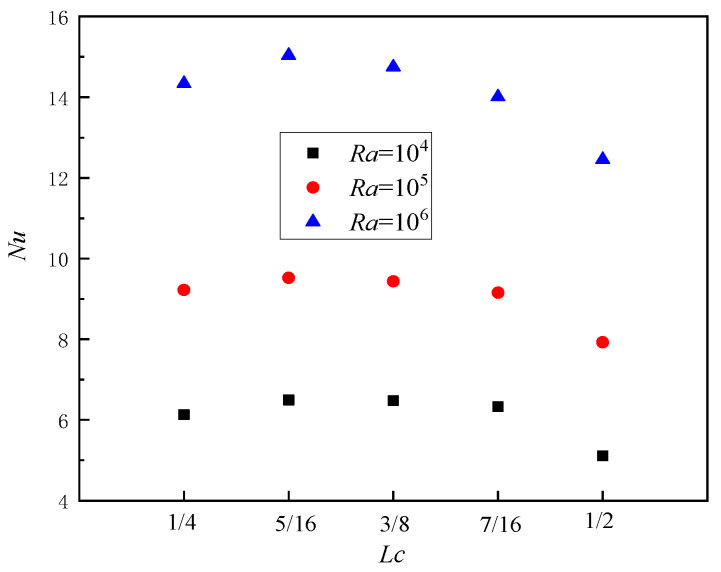
Local *Nu* in the square cavity varies with *Ra* (*A* = 0.1, *n* = π).

Even with an expanded range in *Ra* between 10^4^ and 10^6^, the variation trend of local *Nu* numbers remains consistent when *A* = 0.1 and *n* = π, as depicted in Figure 10. The *Nu* attains its maximum value near *L_c_* = 5/16, followed by a rapid decline after *L_c_* exceeds 7/16. Moreover, for the same initial position *L_c_*, an increase in the *Ra* leads to a corresponding augmentation of the local *Nu* number within the square cavity, thereby enhancing convective heat transfer effects.

### 3.4. Influence of Angular Velocity n of Sinusoidal-Temperature-Distributed Heat Source on Convective Heat Transfer Characteristics

The present section investigates the influence of angular velocity n of a non-uniform heat source on convective heat transfer in a square cavity. In this section, the angular velocity *n* is chosen as an integer multiple of π, ranging from π to 16π, in order to ensure that the heat source component encompasses at least one complete half of a sine wave. The initial position of the heat source is determined at *L_c_* = 1/4, the length of the heat source is *L_h_* = 1/2, and the temperature distribution is *T* = (1 − *A*) + *Asin*(*nx* + *θ*), where θ = 0.

**Figure 11:**
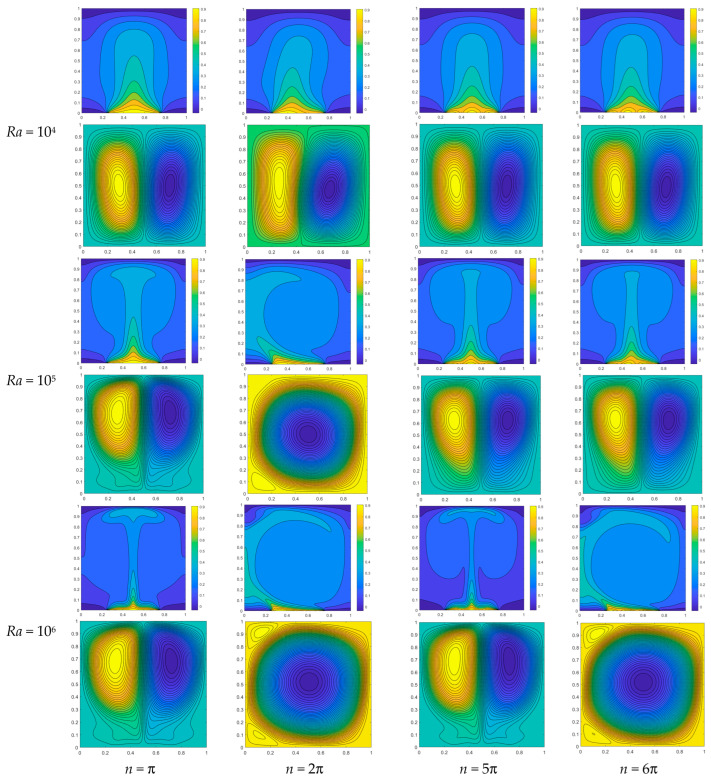
Temperature distribution (**up**) and streamline distribution (**bottom**) under different *n* and *Ra* (*A* = 0.1).

This section initially examines the impact of angular velocity on convective heat transfer in a square cavity with varying Rayleigh numbers, while maintaining *A* = 0.1. When *Ra* = 10^4^ and *n* = π, the heat source exhibits a fully distributed sinusoidal half wave, resulting in a symmetrical isotherm distribution within the temperature field. Consequently, two symmetric main vortices are generated in the flow field. When *n* = 2π, the temperature distribution of the heat source exhibits a complete sine wave pattern, with higher temperatures on the left side and lower temperatures on the right side. Consequently, there is a larger temperature gradient on the left side accompanied by lower density, while the vortex on the right side gradually displaces or compresses the vortex on the left side. As *n* continues to increase up to 5π, the temperature distribution of the heat source region exhibits symmetrical characteristics once again, while maintaining left–right symmetry in the two primary vortices within the flow field. Upon reaching 6π, an increase in n leads to the reappearance of temperature asymmetry in the heat source. However, compared to the case when *n* = 2π, the asymmetry in temperature distribution is less pronounced. The temperature and flow fields will exhibit increased instability at *Ra* = 10^5^, primarily due to the enhanced dominance of buoyancy force over viscous force. When *n* = π, the temperature field and the flow field still exhibit symmetry; however, due to the influence of buoyancy, there is a displacement in the vortex position of the two primary vortices. At *n* = 2π, the asymmetry in heat source temperature exerts a significant impact on the flow field, with the vortex on the right completely dominating it. When *n* increases to 6π, the temperature distribution of the heat source becomes asymmetric once again. However, due to the constant length of the heat source, an increase in *n* leads to a greater number of sine waves distributed within it, resulting in a more uniform temperature distribution. Consequently, as depicted in Figure 11, two primary vortices reappear in the flow field; nevertheless, owing to the influence of temperature heterogeneity, the right vortex continues to exert pressure on the left vortex. As *Ra* = 10^6^, the dominance of buoyancy over viscosity continues to increase, thereby further enhancing the impact of temperature inhomogeneity on the flow field. As depicted in the figure, when n is π and 5π, the heat source exhibits a symmetric temperature distribution, resulting in two symmetric primary vortices within the flow field. However, as n reaches 6π, an independent primary vortex emerges while no secondary vortices of comparable magnitude appear.

**Figure 12:**
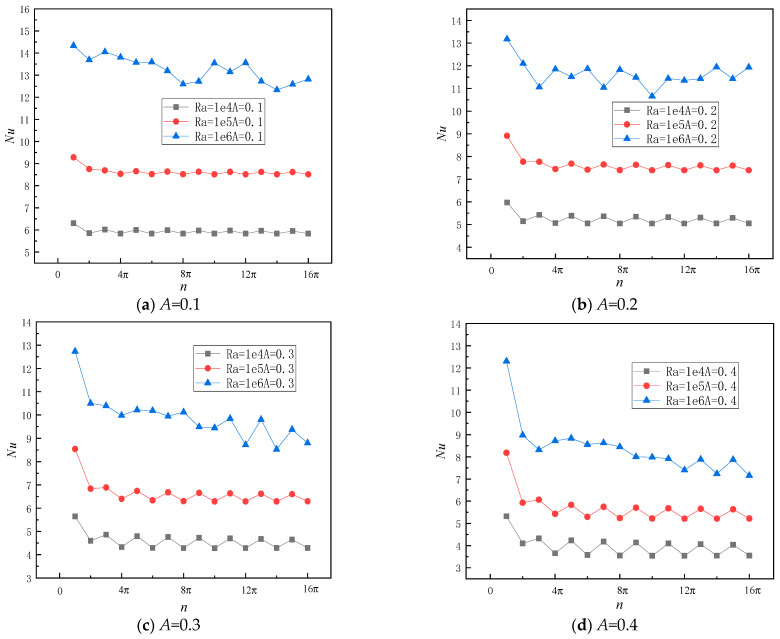
Local *Nu* varies with the angular velocity of sinusoidal-temperature-distributed heat source under different Rayleigh numbers.

Furthermore, this section examines the impact of angular velocity n on convective heat transfer characteristics at varying amplitudes. As depicted in Figure 12a, for *A* = 0.1, *Ra* = 10^4^ and *Ra* = 10^5^, the maximum value of *Nu* is observed at *n* = π. Subsequently, as the angular velocity increases, there is an overall decreasing trend in *Nu*. Notably, when *n* represents an odd multiple of π, a higher *Nu* is obtained, whereas for even multiples of π, *Nu* tends to be smaller. When *Ra* = 10^4^ and *Ra* = 10^5^, *Nu* after *n* exhibits minimal fluctuation, with a marginal difference of 3.1% and 2.8%, respectively. When *Ra* = 10^6^, there is no discernible regular fluctuation trend; however, the magnitude of the change in *Nu* remains consistently close to 10.7%. With the increase in amplitude *A*, *Nu* exhibits a consistent downward fluctuation trend with varying *n* under *Ra* = 10^4^ and *Ra* = 10^5^; however, this trend becomes more pronounced. Simultaneously, when *Ra* = 10^6^ and amplitude *A* varies between 0.3 and 0.4, an overall downward trend becomes evident as *n* increases to approximately 11π, as shown in Figure 12c,d. This is attributed to the increase in *n*, which leads to a higher number of sine waves in the heat source section and subsequently reduces temperature heterogeneity. Notably, when *n* is an odd multiple of π, the temperature distribution within the heat source section becomes symmetrically centered, resembling a constant temperature heat source.

### 3.5. Application of Machine Learning to the Natural Convection of Sinusoidal-Temperature-Distributed Heat Sources

The heat transfer performance of the lower cavity under a specific operational condition is rapidly predicted in this section. Therefore, the BP algorithm is employed to establish the correlation between the key parameters (*Ra*, *A*, *n*) and *Nu* that governs the convective heat transfer performance of the square cavity.

**Figure 13:**
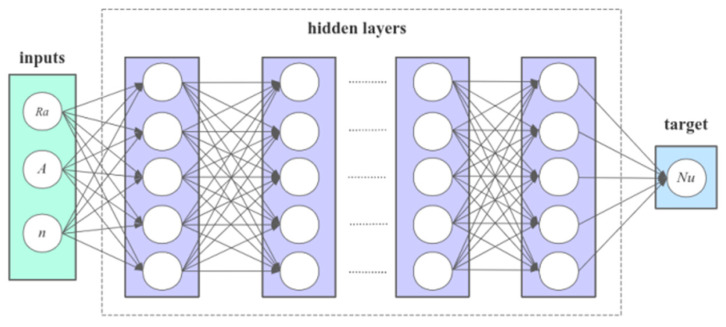
Schematic diagram of neural network.

The input parameters (*Ra*, *A*, *n*) are utilized in this section to generate the corresponding *Nu* through LBM under specific working conditions. Consequently, a dataset comprising 372 groups is generated. The dataset is then randomly shuffled to mitigate the risk of overfitting in the training model, subsequently partitioned into three subsets: the training data set, validation data set, and test data set, with proportions of 80%, 10%, and 10%, respectively. The neural network model structure employed in this section comprises an input layer, 10 hidden layers, and an output layer, as depicted in Figure 13.

**Figure 14:**
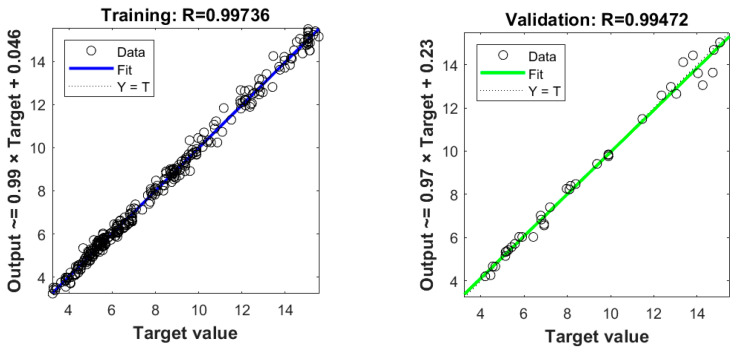

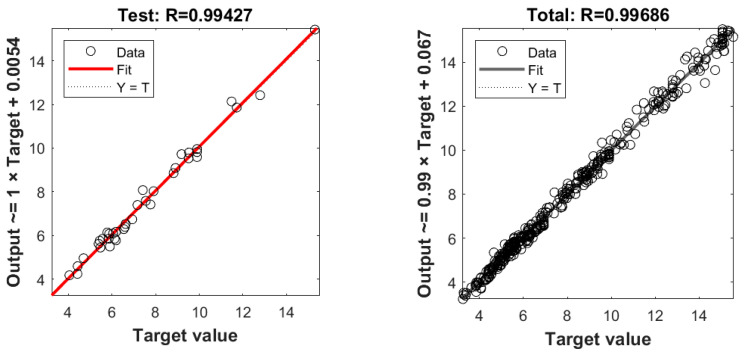
Plots of the fitted *Nu* (output) versus the exact *Nu* (target value).

The Levenberg–Marquardt backpropagation algorithm, known for its second-order training speed, is employed in the training process of the neural network. The training results are illustrated in Figure 14. After the training, the R values of the training data, verification data, and test data all exceed 0.99, demonstrating high accuracy. Moreover, the predicted values exhibit excellent agreement with the simulation results obtained from the lattice Boltzmann method.

**Figure 15:**
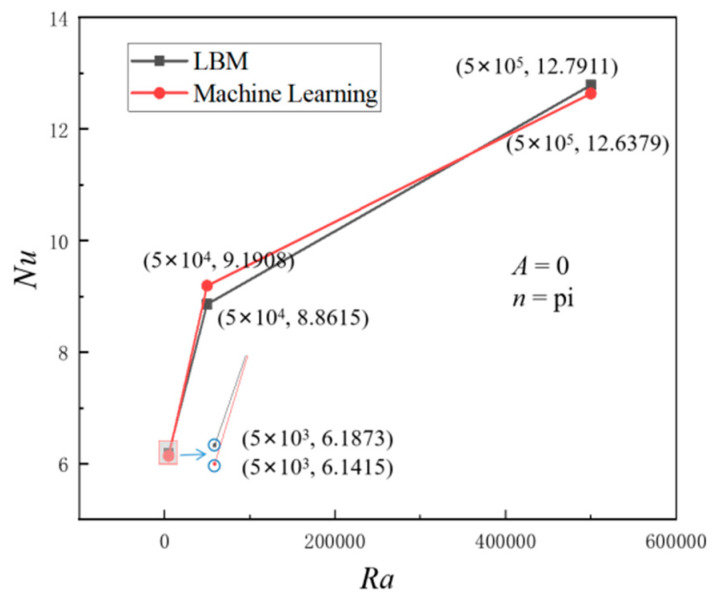
Comparative analysis of *Nu* prediction results and LBM simulation results under different *Ra* (*A* = 0 *n* = π).

Therefore, this section applies the model to novel working conditions, that is, conditions that have not been encountered previously, in order to predict the heat transfer characteristics under corresponding circumstances and compare them with the LBM simulation results obtained under identical operating conditions. The comparison between machine learning prediction results and LBM simulation results is illustrated in Figure 15, for the case where *A* = 0 and *n* = π, with *Ra* ranging from 5 × 10^3^ to 5 × 10^5^.

The figure demonstrates a strong agreement between the predicted results and the LBM simulation results, with a relative error of less than 4%, falling well within the acceptable range of accuracy. Therefore, the model’s generalization performance and accuracy are further validated, providing valuable insights for the application of machine learning in the field of heat transfer.

## 4. Conclusions

The lattice Boltzmann method (LBM) is employed in this study to simulate the natural convection phenomenon occurring due to non-uniform heating at the bottom of a square cavity. The primary focus lies on analyzing the impact of heat transfer on the phase angle, initial position, and angular velocity of the heat source. Additionally, the present study establishes a machine learning-based predictive model for the characterization of heat transfer properties. Some suggestions can be put forward for the heat exchange equipment involving non-uniform heating at the bottom of the square cavity. The results are as follows:

In the phase angle range from 0 to π, *Nu* basically shows a decreasing trend with an increase in the phase angle. The decline in *Nu* at an accelerated rate is consistently observed when the phase angle reaches 4π/16. The corresponding *Nu* decreases as the amplitude increases at the same phase angle. The *Nu* of the large amplitude value *A* exhibits a more pronounced decrease with an increase in the phase angle.

The initial position of the sinusoidal-temperature-distributed heat source *L_c_* significantly impacts the convective heat transfer in the cavity. When *L_c_* reaches 7/16, *Nu* exhibits a discernible decrease by approximately 13%, thereby enabling the identification of the optimal location for the initial heat source.

Modifying the angular velocity of a sinusoidal-temperature-distributed heat source exerts a discernible impact on the heat transfer characteristics within the square cavity. The optimal overall heat transfer effect is achieved when *n* equals π. When the angular velocity is an even multiple of π, the local *Nu* decreases, whereas when it is an odd multiple of π, the local *Nu* increases.

In the current work, the integration of machine learning and the lattice Boltzmann method (LBM) enables the rapid and accurate prediction of *Nu* in natural convection processes, thereby offering valuable insights for the application of machine learning in the field of heat transfer.

## Figures and Tables

**Table 1:** Comparison of simulation results (*Pr* = 0.71, *Ra* = 2.02 × 10^5^) with the literature under different grid distributions.

Grid Distribution	*Nu*	*Nu* Relative Error (%)
$Nu=0.5478Ra^{0.1998}$	6.2897	
140 × 140	5.8980	6.22
180 × 180	6.0454	3.88
200 × 200	6.0969	3.07

## Data Availability

The data presented in this study are available on request from the corresponding author.

## Nomenclature

g	Acceleration of gravity ($m\cdot s^{-2}$)
*p*	Fluid pressure (Pa)
*F*	Force term (N)
*Nu*	Nusselt number
*Pr*	Prantdl number
*Ra*	Rayleigh number
*T*	Temperature (K)
*T′*	Dimensionless temperature
***V***	Macroscopic velocity ($m\cdot s^{-1}$)
***V′***	Dimensionless velocity in a square cavity
*X*	The horizontal direction of the Cartesian coordinates
*Y*	The vertical direction of the Cartesian coordinates
*A*	The amplitude of a sinusoidal-temperature-distributed heat source
*L*	The dimensionless length of a square cavity
*n*	The angular velocity
*t*	Time (s)
*t′*	Dimensionless time
*f*	Velocity distribution function
*feq*	Equilibrium velocity distribution function
*c*	Lattice velocity
*cs*	Sound velocity
*a*	Lattice direction
*H*	Temperature distribution function
*Heq*	Temperature equilibrium distribution function
*w*	The equilibrium distribution weight
Greek symbols	
θ	Phase Angle of sinusoidal-temperature-distributed heat source
ρ	Density ($g\cdot {\mathrm{cm}}^{-3}$)
ω	Relaxation factor
α	Thermal diffusion coefficient(m^2^·s^−1^)
β	Coefficient of volume expansion(K^−1^)
μ	Dynamic viscosity (Pa·s)
Subscripts	
*c*	Low-temperature heat source
*h*	High-temperature heat source
*m*	Motion lattice
*s*	Thermal lattice

## References

[1] ElSherbiny S.M., Raithby G.D., Hollands K.G.T. (1982). Heat Transfer by Natural Convection Across Vertical and Inclined Air Layers. J. Heat Transf..

[2] Lage J.L., Lim J.S., Bejan A. (1992). Natural Convection With Radiation in a Cavity With Open Top End. J. Heat Transf..

[3] Ferdows M., Liu D. (2018). Natural convective flow of a magneto-micropolar fluid along a vertical plate. Propuls. Power Res..

[4] Kiš P., Herwig H. (2012). The near wall physics and wall functions for turbulent natural convection. Int. J. Heat Mass Tran..

[5] Zhang D., Ding B., Zhu C., Gong L. (2022). Enhancement of Natural Convection for Cooling Active Antenna Unit Device in 5G Base Station. J. Therm. Sci..

[6] Luo W.J., Yang R.J. (2007). Multiple fluid flow and heat transfer solutions in a two-sided lid-driven cavity. Int. J. Heat Mass Tran..

[7] Wei Y., Dou H., Wang Z., Qian Y., Yan W. (2016). Simulations of natural convection heat transfer in an enclosure at different Rayleigh number using lattice Boltzmann method. Comput. Fluids.

[8] Javaherdeh K., Moslemi M., Shahbazi M. (2017). Natural convection of nanofluid in a wavy cavity in the presence of magnetic field on variable heat surface temperature. J. Mech. Sci. Technol..

[9] Zhu J., Hou J., Gao D., Lin F., Chen W., Lu S. (2018). Simulation of Natural Convection in an Inclined Square Cavity Based on Lattice Boltzmann Method. J. Nanjing Norm. Univ. (Eng. Technol. Ed.).

[10] Huelsz G., Rechtman R. (2013). Heat transfer due to natural convection in an inclined square cavity using the lattice Boltzmann equation method. Int. J. Therm. Sci..

[11] Li P., Li W., Zhang Y., Sun J., Wang Z. (2018). Non-orthogonal MRT-LB numerical simulation of natural convection in inclined porous square cavity. J. South China Univ. Technol. (Nat. Sci. Ed.).

[12] Tian Z., Tang Z., Qi C., Chen L., Wang Y. (2022). Natural convection heat transfer characteristics of sinusoidal cavities filled with nanofluids. Colloids Surf. A Physicochem. Eng. Asp..

[13] Uddin M.J., Rasel S.K., Adewole J.K., Al Kalbani K.S. (2022). Finite element simulation on the convective double diffusive water-based copper oxide nanofluid flow in a square cavity having vertical wavy surfaces in presence of hydro-magnetic field. Results Eng..

[14] Jain S., Bhargava R. (2023). Natural convection flow on a bent wavy vertical enclosure filled with power-law nanofluid simulated by Element Free Galerkin method. Math. Comput. Simulat..

[15] Akbarzadeh P., Fardi A.H. (2018). Natural Convection Heat Transfer in 2D and 3D Trapezoidal Enclosures Filled with Nanofluid. J. Appl. Mech. Tech. Phys..

[16] Ren J.Y., Wang Z.Y., Qi R.S., Wu Y.L. (2019). Numerical Simulation of Natural Convection Heat Transfer Characteristics in High Closed Cuboid Cavity. Reneng Dongli Gongcheng J. Eng. Therm. Energy Power.

[17] He Z., Yan W., Zhang K., Yang X., Wei Y. (2017). Simulation of effect of bottom heat source on natural convective heat transfer characteristics in a porous cavity by lattice Boltzmann method. Acta Phys. Sin. Ch. Ed..

[18] Lafdaili Z., El-Hamdani S., Bendou A., Limam K., El-Hafad B. (2021). Numerical Study of the Turbulent Natural Convection of Nanofluids in a Partially Heated Cubic Cavity. Therm. Sci..

[19] Turner B.J.S. (1973). Buoyancy Effects in Fluids.

[20] Guo Z.G.Z., Zheng C.Z.C.H. (2008). Analysis of lattice Boltzmann equation for microscale gas flows: Relaxation times, boundary conditions and the Knudsen layer. Int. J. Comput. Fluid D.

[21] Bhatnagar P.L., Krook E.P.G.A. (1954). A model for collision processes in gases. I. Small amplitude processes in charged and neutral one-component systems. Phys. Rev..

[22] Qian Y.H., D’Humières D., Lallemand P. (1992). Lattice BGK Models for Navier-Stokes Equation. Europhys. Lett..

[23] Corvaro F., Paroncini M. (2008). A numerical and experimental analysis on the natural convective heat transfer of a small heating strip located on the floor of a square cavity. Appl. Therm. Eng..

[24] Shao M., Yan W., He Z. (2019). Numerical Study on Natural Convective Heat Transfer Characteristics in a Porous Cavity Heated from Bottom. J. Eng. Thermophys. Rus..

